# Bacterial lipopolysaccharide as negative predictor of gemcitabine efficacy in advanced pancreatic cancer – translational results from the AIO-PK0104 Phase 3 study

**DOI:** 10.1038/s41416-020-01029-7

**Published:** 2020-08-24

**Authors:** Michael Guenther, Michael Haas, Volker Heinemann, Stephan Kruger, Christoph Benedikt Westphalen, Michael von Bergwelt-Baildon, Julia Mayerle, Jens Werner, Thomas Kirchner, Stefan Boeck, Steffen Ormanns

**Affiliations:** 1 Institute of Pathology, Faculty of Medicine, Ludwig-Maximilians-University, Munich, Germany; 2 Department of Internal Medicine III, Grosshadern University Hospital, Ludwig-Maximilians-University, Munich, Germany; 3 German Cancer Consortium (DKTK), partner site Munich, Munich, Germany; 4 Department of Internal Medicine II, Grosshadern University Hospital, Ludwig-Maximilians-University, Munich, Germany; 5 Department of General, Visceral and Transplant Surgery, Ludwig-Maximilians-University, Munich, Germany

**Keywords:** Pancreatic cancer, Predictive markers, Cancer microenvironment, Pancreatic cancer

## Abstract

**Background:**

Gram-negative bacteria mediated gemcitabine resistance in pre-clinical models. We determined if intratumoural lipopolysaccharide (LPS) detection by immunohistochemistry is associated with outcome in advanced pancreatic ductal adenocarcinoma (PDAC) treated with gemcitabine and non-gemcitabine containing 1st-line chemotherapy.

**Methods:**

We examined LPS on tumour tissue from 130 patients treated within the randomised AIO-PK0104 trial and a validation cohort (*n* = 113) and analysed the association of LPS detection to patient outcome according to treatment subgroups.

**Results:**

In 24% of samples from the AIO-PK0104 study LPS was detected; in LPS-positive patients median OS was 4.4 months, compared to 7.3 months with LPS negative tumours (HR 1.732, *p* = 0.010). A difference in OS was detected in 1st-line gemcitabine-treated patients (*n* = 71; HR 2.377, *p* = 0.002), but not in the non-gemcitabine treatment subgroup (*n* = 59; HR 1.275, *p* = 0.478). Within the validation cohort, the LPS positivity rate was 23%, and LPS detection was correlated with impaired OS in the gemcitabine subgroup (*n* = 94; HR 1.993, *p* = 0.008) whereas no difference in OS was observed in the non-gemcitabine subgroup (*n* = 19; HR 2.596, *p* = 0.219).

**Conclusions:**

The detection of intratumoural LPS as surrogate marker for gram-negative bacterial colonisation may serve as a negative predictor for gemcitabine efficacy in advanced PDAC.

**Clinical trial registry:**

The Clinical trial registry identifier is NCT00440167.

## Background

The majority of patients with pancreatic ductal adenocarcinoma (PDAC) is diagnosed in an advanced stage, resulting in mortality rates almost equal to incidence rates.^1^ Palliative chemotherapy is considered as an international standard of care for these patients; however, relevant predictive biomarkers for rational treatment decisions are still lacking.^2^ Increasing evidence indicates a crucial role for the human microbiome in solid malignancies, especially in gastrointestinal tumours which are in close contact to dense microbial colonisation.^3,4^ Importantly, intratumoural bacteria have been shown to promote tumorigenesis^5^ and to confer resistance to gemcitabine in human adenocarcinoma.^6,7^ As reported recently, intratumoural bacteria can metabolise gemcitabine (2′,2′-difluorodeoxycytidine) into its inactive form 2′,2′-difluorodeoxyuridine; in a pre-clinical model, gemcitabine depletion was dependent on the expression of a long isoform of the bacterial enzyme cytidine deaminase (CDD_L_) commonly found in gram-negative bacteria.^7^ Bacterial lipopolysaccharide is a major component of the cell wall of gram-negative bacteria and can easily be detected by immunohistochemistry.^7^

In the present study, we report the results of immunohistochemical detection of intratumoural LPS as a surrogate marker of gram-negative bacterial colonisation in the tumour tissue of 243 patients with advanced PDAC from two independent study cohorts and the correlation of intratumoural LPS detection to patient outcome according to the applied 1st-line chemotherapy regimen (gemcitabine vs non-gemcitabine containing).

## Methods

### Patients and tumour samples

Archival tumour material was derived from 130 advanced PDAC patients treated within the randomised Phase 3 study AIO-PK0104 (NCT00440167), that compared a 1st-line treatment with gemcitabine + erlotinib versus capecitabine + erlotinib (with the option of a cross-over to the comparator cytotoxic drug in the 2nd-line setting).^8^ A validation of the LPS results from AIO-PK0104 was performed on 113 patient samples derived from a previously reported prospective biomarker trial,^9^ in which the 1st-line chemotherapy regimens were as follows: 40 patients received gemcitabine monotherapy, 29 patients gemcitabine and cisplatin, 10 patients gemcitabine and oxaliplatin, 14 gemcitabine and capecitabine, 11 patients single-agent capecitabine, 7 patients capecitabine and oxaliplatin, one patient received FOLFOX-6 and one patient received gemcitabine and 5-FU. Both studies had approval of the local ethics committee and were conducted according to GCP/ICH.

### LPS immunohistochemistry

Intratumoural bacterial LPS was detected immunohistochemically on formalin fixed paraffin embedded (FFPE) tumour material as described previously^10^ using a monoclonal mouse anti-LPS antibody detecting the core bacterial LPS antigen (clone WN1 222-5, Hycult Biotech, Uden, The Netherlands) at a 1:800 dilution. Tumours from 133 patients were included in a tissue microarray (TMA) as three cores of one mm diameter each (71 samples from the AIO-PK0104 cohort, 62 samples from the validation cohort), whereas biopsies from 110 patients were stained as whole-mount tissue sections (59 samples from the AIO-PK0104 cohort, 51 samples from the validation cohort). Normal human colonic mucosa, which physiologically carries high amounts of gram-negative bacteria and normal human liver tissue, which physiologically may show high amounts of LPS, were used as positive controls in each staining run (Supplementary Fig. S1A, B). Of note, normal adjacent pancreatic tissue did not show LPS signals nor unspecific staining in acinar cells (Fig. S1C). On TMA embedded tumour tissue, cases showing strong signals in at least one TMA core or weak and single signals in at least two cores were evaluated as positive, whereas all others were evaluated as negative. On whole-mount tissue sections, the presence of definitive LPS signals throughout the tumour tissue was evaluated as positive, whereas complete absence of signal was evaluated as negative. All slides were read and scored by two researchers (M.G. and S.O.), blinded to the patient outcome and discrepant cases were discussed until agreement was reached. Microphotographs were acquired as described previously.^10^

### Statistical analyses

Progression free survival (PFS) and overall survival (OS) were calculated from the initiation of 1st-line chemotherapy to the occurrence of an event (progress, death). The correlation of tumour or patient characteristics to PFS and OS was calculated using the Kaplan–Meier method. Hazard ratios were estimated by Cox proportional hazards regression. Correlation of patient or sample characteristics was assessed using cross tabulations and two sided *χ*^2^-tests. Statistical analyses were run on SPSS software (IBM, Ehningen, Germany).

## Results

### Patient characteristics

In the overall population (*n* = 243) median patient age was 62 years; 145 patients were male. On therapy initiation, 23 patients from the AIO-PK0104 trial and 10 patients from the validation cohort had locally advanced disease, whereas all others had distant metastases. Both cohorts were comparable in terms of patient age, sex, KPS groups as well as tissue sample origin and tissue modality (TMA vs whole slide). In the AIO-PK0104 cohort, we detected more locally advanced cases, higher grade tumours and less lung metastases. Related to the trial design, more patients received non-gemcitabine-based 1st-line chemotherapy in the AIO-PK0104 cohort (Table S1). All clinicopathologic patient characteristics and associated survival times of each subgroup are summarised in Table S2.

### LPS detection and clinico-pathological patient characteristics

**Fig. 1:**
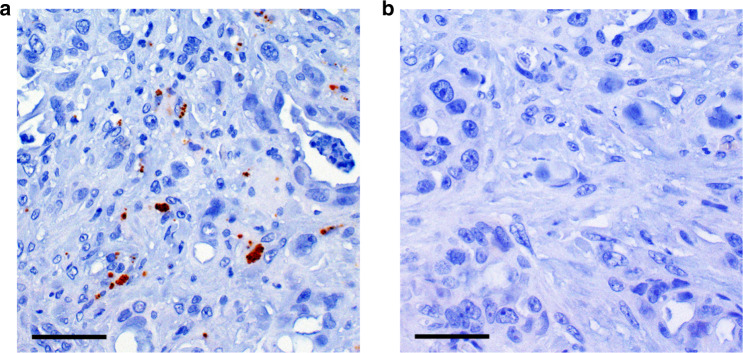
Intratumoural LPS can be detected in advanced PDAC tumour samples. Immunohistochemical detection of bacterial lipopolysaccharide (LPS) in pancreatic cancer. Exemplary cases of LPS positive (**a**) and LPS negative (**b**) pancreatic cancer samples. 200-fold magnification. Scale bars indicate 50 µm.

Using immunohistochemistry, bacterial LPS could reliably be detected in 24.5% of all PDAC samples (Fig. 1). To preclude potential sampling errors due to the use of TMA embedded tissue, we compared the staining result in whole-mount tissue sections and the TMA cores of 10 exemplary positive and 15 exemplary negative cases from both cohorts and found complete consistency (Table S3 and Fig. S2A, B). Interestingly, we detected a significantly higher rate of LPS positivity in tumour samples which were derived from metastatic sites compared to primary tumour tissue samples (36.5% vs 9.4%, *p* < 0.001, Table 1). As expected in a cohort of advanced PDAC patients, tissue samples from liver metastases constituted the largest group (96 of 243 samples) and showed a significantly higher LPS positivity rate than tissue from other metastatic sites or primary tumours (55.2% vs 9.3%, *p* < 0.001, Table 1). In the subgroup in which only primary tumour samples were examined, no statistically significant association of primary tumour location and LPS detection was found (*p* = 0.484, Table S4). Moreover, we found no statistically significant association between LPS detection and patient characteristics such as gender, age group or KPS group (Table 1).

**Table 1:** Association of clinicopathologic variables and LPS detection in the entire study cohort.

	LPS			*p*-value (*χ*^2^-test)
	−	+	Total	
*Primary tumour location*				
Unknown	17 (73.9)	6 (26.1)	23 (9.5)	0.617
Pancreatic head	103 (74.1)	36 (25.9)	139 (57.2)	
Pancreatic body	39 (83.0)	8 (17.0)	47 (19.3)	
Pancreatic tail	27 (79.4)	7 (20.6)	34 (14.0)	
Total	186 (76.5)	57 (23.5)	243 (100.0)	
*Metastasis at therapy initiation*				
Lung	5 (83.3)	1 (16.7)	6 (2.5)	0.023
Liver	65 (67.7)	31 (32.3)	96 (39.5)	
Peritonuem	12 (100.0)	0 (0.0)	12 (4.9)	
Other	16 (69.6)	7 (30.4)	23 (9.5)	
None	32 (97.0)	1 (3.0)	33 (13.6)	
Liver and peritoneum	17 (94.4)	1 (5.6)	18 (7.4)	
Lung and liver	6 (54.5)	5 (45.5)	11 (4.5)	
Liver and bone	1 (100.0)	0 (0.0)	1 (0.4)	
Lung and abdominal lymph nodes	3 (100.0)	0 (0.0)	3 (1.3)	
Liver and lymph nodes	16 (72.7)	6 (27.3)	22 (9.1)	
Liver, lung and lymph nodes	3 (75.0)	1 (25.0)	4 (1.6)	
Lymph nodes	8 (72.7)	3 (27.3)	11 (4.5)	
Liver and adrenal gland	1 (50.0)	1 (50.0)	2 (0.8)	
Lung, liver and bone	1 (100.0)	0 (0.0)	1 (0.4)	
Total	186 (76.5)	57 (23.5)	243 (100.0)	
*Tissue origin*				
Hepatic metastasis	59 (57.3)	44 (42.7)	103 (42.4)	0.000
Primary tumor	106 (90.6)	11 (9.4)	117 (48.1)	
Peritoneal metastasis	8 (100.0)	0 (0.0)	8 (3.3)	
Lung metastasis	5 (83.3)	1 (16.7)	6 (2.5)	
Lymph node	4 (100.0)	0 (0.0)	4 (1.6)	
Other/unknown	4 (80.0)	1 (20.0)	5 (2.1)	
Total	186 (76.5)	57 (23.5)	243 (100.0)	
*Age group*				
≤60	75 (73.5)	27 (26.4)	102 (42.0)	0.346
>60	111 (77.6)	30 (22.4)	141 (58.0)	
Total	186 (76.5)	57 (23.5)	243 (100.0)	
*Gender*				
Male	112 (77.2)	33 (22.8)	145 (59.7)	0.755
Female	74 (75.5)	24 (24.5)	98 (40.3)	
Total	186 (76.5)	57 (23.5)	243 (100.0)	
*KPS group*				
≤80	75 (72.8)	28 (27.2)	103 (42.7)	0.210
> 80	110 (79.7)	28 (20.3)	138 (57.3)	
Total	185 (76.8)	56 (23.2)	241 (100.0)	
*CTX type*				
Non-gemcitabine 1st line subgroup	64 (82.1)	14 (17.9)	78 (32.1)	0.164
Gemcitabine 1st line subgroup	122 (73.9)	43 (26.1)	165 (67.9)	
Total	186 (76.5)	57 (23.5)	243 (100.0)	
*Disease stage at therapy initiation*				
Locally advanced	32 (97.0)	1 (3.0)	33 (13.6)	0.003
Metastatic	154 (73.3)	56 (26.7)	210 (86.4)	
Total	186 (76.5)	57 (23.5)	243 (100.0)	
*Grade group*				
G1-G2	75 (84.3)	14 (15.7)	89 (36.6)	0.031
G3-G4	111 (72.1)	43 (27.9)	154 (63.4)	
Total	186 (76.5)	57 (23.5)	243 (100.0)	

### Intratumoural LPS detection is associated with inferior survival in gemcitabine-treated PDAC patients

**Fig. 2:**
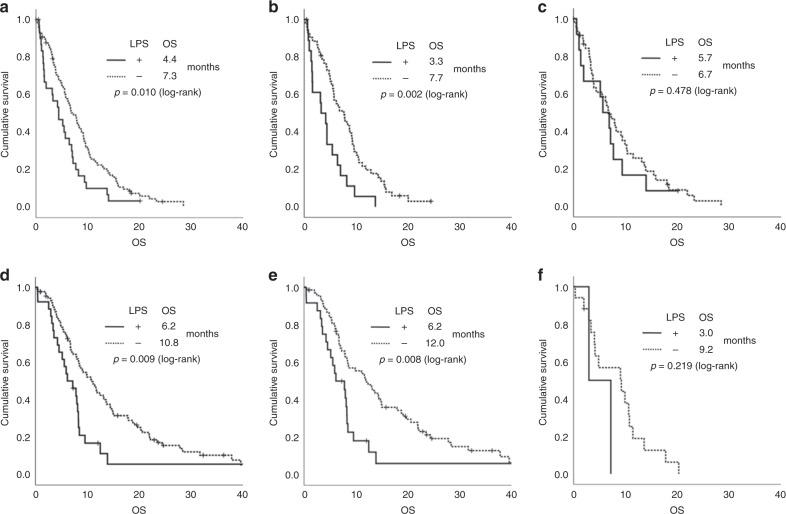
Intratumoural LPS is associated with inferior OS in gemcitabine-treated PDAC patients. Overall survival (OS) of each patient subgroup according to intratumoural LPS detection. Kaplan–Meier plots of OS in the **a** AIO-PK0104 overall cohort. **b** 1st-line gemcitabine subgroup of the AIO-PK0104 trial population. **c** 1st-line capecitabine subgroup of the AIO-PK0104 trial population. **d** Validation overall cohort. **e** 1st-line gemcitabine subgroup of the validation cohort. **f** 1st-line non-gemcitabine subgroup of the validation cohort. Crossed lines indicate censored cases.

We detected bacterial LPS in the tumour material of 24% of the 130 patients from the AIO-PK0104 study, which was significantly associated to inferior OS (4.4 vs 7.3 months, HR 1.732, *p* = 0.010, Fig. 2a). Intratumoural LPS was significantly associated with a shorter OS in the 1st-line gemcitabine randomisation arm (3.3 *vs* 7.7 months, HR 2.377, *p* = 0.002, Fig. 2b), whereas no such difference in the 1st-line capecitabine arm was detected (5.7 vs 6.7 months, HR 1.275, *p* = 0.478, Fig. 2c). All 113 patients selected for the validation cohort received erlotinib-free, either gemcitabine-based or non-gemcitabine-based 1st-line chemotherapy regimens^9^; 23% of these tumour samples were LPS positive, which again was significantly associated to shorter patient PFS (4.1 vs 7.8 months, HR 1.760, *p* = 0.028) and OS (6.2 vs 10.8 months, HR 1.880, *p* = 0.009, Fig. 2d). In subgroup analyses, the negative impact of LPS detection on either PFS (HR 2.051, *p* = 0.010) or OS (HR 1.993, *p* = 0.008, Fig. 2e) was restricted to the gemcitabine subgroup, whereas no difference in PFS (HR 1.292, *p* = 0.742) or OS (HR 2.596, *p* = 0.219, Fig. 2f) was detected for the non-gemcitabine subgroup with regard to LPS status, respectively (Table 2).

**Table 2:** Patient prognosis according to LPS detection in each patient subgroup.

	LPS	*n*	%	OS (months)	*p* (log-rank)	HR	95% CI	PFS (months)	*p* (log-rank)	HR	95% CI
*All patients*											
Total	+	57	24.5	5.6	0.000	1805	1.312–2.482	3.5	0.034	1413	1.024–1.951
	−	186	76.5	8.5				4.0			
AIO-PK0104	+	31	23.9	4.4	0.010	1732	1.133–2.649	1.8	0.249	1278	0.840–1.944
	−	99	76.2	7.3				2.8			
Validation cohort	+	26	23.0	6.2	0.009	1880	1.161–3.045	4.1	0.028	1760	1.055–2.936
	−	87	77.0	10.8				7.8			
*1st line gemcitabine subgroup*											
Total	+	43	35.2	5.5	0.000	2.081	1.425–3.038	3.7	0.005	1.728	1.175–2.542
	−	122	64.8	8.8				6.1			
AIO-PK0104	+	19	26.8	3.3	0.002	2.377	1.353–4.178	3.2	0.062	1.681	0.968–2.919
	−	52	73.2	7.7				3.6			
Validation cohort	+	24	25.5	6.2	0.008	1.993	1.190– 3.338	4.7	0.010	2.051	1.177–3.573
	−	70	74.5	12.0				9.2			
*1st line non-gemcitabine subgroup*											
Total	+	14	18.0	5.7	0.306	1.373	0.747– 2.526	2.2	0.955	1.018	0.554–1.868
	−	64	82.0	7.3				2.2			
AIO-PK0104	+	12	20.3	5.7	0.478	1.275	0.651– 2.497	1.7	0.829	0.929	0.475–1.817
	−	47	79.7	6.7				2.2			
Validation cohort	+	2	10.5	3.0	0.219	2.596	0.536– 12.580	2.2	0.742	1.292	0.278–6.014
	−	17	89.5	9.2				2.7			

Median times of overall survival (OS) and progression free survival (PFS) according to intratumoural LPS detection in each study subgroup as well as corresponding hazard ratios (HR) and 95% confidential intervals (95% CI).

In Cox multivariate regression analyses adjusting for the parameters which were statistically significant prognosticators in univariate analyses (tumour grade, KPS and disease stage at chemotherapy initiation), we confirmed LPS detection as independent negative predictor for both PFS and OS in the gemcitabine 1^st^-line chemotherapy subgroup (Table 3).

**Table 3:** Intratumoural LPS is an independent negative prognostic biomarker in gemcitabine-treated PDAC patients.

	OS			
	parameter	*p* (Cox)	HR	95% CI
All cases	Grade group	0.002	1.592	1.194–2.122
	KPS group	0.000	0.601	0.455–0.793
	LPS	0.002	1.664	1.203–2.302
Gemcitabine 1st line subgroup	Grade group	0.000	1.977	1.401–2.790
	Disease stage	0.029	1.877	1.065–3.306
	LPS	0.002	1.872	1.267–2.765
Non-gemcitabine 1st line subgroup	KPS group	0.000	0.401	0.246–0.654

	PFS			
	Parameter	*p* (Cox)	HR	95% CI
All cases	Grade group	0.002	1.610	1.187–2.186
	KPS group	0.002	0.637	0.476–0.853
	CTX type	0.000	0.456	0.335–0.620
Gemcitabine 1st line subgroup	LPS	0.009	1.674	1.136–2.467
	Grade group	0.001	1.838	1.275–2.648
Non-gemcitabine 1st line subgroup	KPS group	0.002	0.435	0.258–0.733

Cox regression analyses in the indicated patient subgroups for overall survival (OS) and (PFS), adjusting for tumour grade, KPS group, type of 1st-line palliative chemotherapy and disease stage at 1st-line therapy initiation.

KPS: Karnofsky performance status; tumour grade: high grade (G3-G4) vs low grade (G1-G2) differentiation; CTX-type: type of 1st-line chemotherapy (gemcitabine-based vs non-gemcitabine based); disease stage: disease stage at 1st-line therapy initiation (metastatic vs locally advanced).

To preclude a potential impact of the imbalance of LPS positivity in metastatic and primary tumour tissue, we compared OS and PFS according to LPS detection in each subgroup and obtained similar results as in the overall cohorts (Table S5). Similarly, we tested whether tissue modality (whole-mount sections vs. TMA) affected the association of LPS detection and patient outcome. Although we detected significantly different positivity rates in both subgroups, LPS positivity remained a negative predictor for outcome in gemcitabine-based 1^st^-line treated patients, whereas we detected no significant differences in non-gemcitabine-treated patients (Table S6).

### Intratumoural LPS detection is associated with inferior tumour therapy response in gemcitabine-treated PDAC patients

From 110 patients treated within the AIO-PK0104 study, objective tumour response data (a secondary trial endpoint) was available, but we did not detect a significant correlation of LPS positivity and tumour response estimated radiologically by RECIST (Table S7). As the reliability of radiologic assessment of tumour response in PDAC remains a matter of debate,^11^ we additionally assessed the biochemical treatment response by CA19-9 serum levels. Data on baseline CA19-9 levels and after the first or third chemotherapy cycle (eight weeks for the gemcitabine 1st-line subgroup, nine weeks for the capecitabine 1st-line subgroup^8^) were available from 95 patients in the AIO-PK0104 cohort. By defining a biochemical response as drop in CA19-9 levels of at least 20% from baseline levels,^12^ only 2 patients with LPS-positive tumours in the gemcitabine 1st-line subgroup (*n* = 54) showed a CA 19-9 response, whereas 10 patients with LPS-positive tumours were non-responders. Within the non-gemcitabine 1st-line subgroup (*n* = 41) no significant association between LPS status and CA 19-9 response was detected (for details see Table 4).

**Table 4:** LPS positivity is associated with a lower biochemical response in gemcitabine-treated patients.

	LPS			
	−	+	Total	*p* (*χ*^2^)
*Gemcitabine 1st line subgroup*				
CA 19-9 non-responder	20	10	30	0.028
CA 19-9 responder	22	2	24	
Total	42	12	54	
*Non-gemcitabine 1st line subgroup*				
CA 19-9 non-responder	20	4	24	0.585
CA19-9 responder	13	4	17	
Total	33	8	41	

Cross-tabs calculation of biochemical treatment response according to LPS status in 95 patients from the AIO-PK0104 cohort, defining a drop in CA19-9 of at least 20% compared to baseline level after the first chemotherapy cycle as ‘CA 19-9 response’.

## Discussion

The impact of the tumour microbiome on patient outcome and therapy response in several human solid malignancies has recently gained increasing attention.^3,13^ In far contrast to other malignancies like colorectal or lung cancer, biomarkers that predict treatment efficacy of specific drugs are still lacking in PDAC. In 2017, Geller et al. reported pre-clinical data suggesting that intratumoural bacteria expressing CDD^L^ mediate tumour resistance to gemcitabine.^7^ However, as to our best knowledge, these data have never been clinically validated yet. As gemcitabine is mainly applied in PDAC, we thought to validate the hypotheses from Geller and co-workers on patient samples from our previously conducted clinical trials in advanced PDAC.^8,9^ As both the AIO-PK0104 cohort as well as the validation cohort contained patients treated with 1st-line gemcitabine and also non-gemcitabine containing chemotherapy, we thought to evaluate a potential predictive role for the occurrence of intratumoural bacteria on gemcitabine efficacy.

Unfortunately, no positivity rates of LPS immunohistochemistry were reported in the study by Geller et al., but the rate of 25% LPS positivity in our overall study population was significantly lower than the rate of 76% ‘bacterial DNA’-positive PDAC samples reported in their study.^7^ However, as their molecular assay detects all kinds of bacteria with superior sensitivity compared to immunohistochemistry and LPS is a cell wall component of gram-negative bacteria only, both figures are not directly comparable. One also might expect higher positivity rates examining whole-mount primary tumour resection specimens—as performed by Geller et al.—compared to biopsy tissue or TMA embedded tumour material. Nevertheless, we precluded a potential bias resulting from tissue modality and confirmed the reliability of TMA embedded tissue to detect intratumoural LPS compared to whole-mount tissue. Moreover, although our study population consisted of patients with advanced PDAC, where histological confirmation is often performed by a percutaneous biopsy of metastases,^9^ we even detected higher positivity rates in metastases than in primary tumours, which was mostly due to more LPS-positive liver metastases. However, intratumoural LPS detection remained a potential negative predictor of gemcitabine efficacy irrespective of the tissue origin subgroup or the modality of the tissue examined (whole-mount sections vs TMA).

Our main results (summarised within Table 2) provide evidence that LPS detection in FFPE tumour samples may serve as a negative predictor for gemcitabine efficacy in advanced PDAC, thereby confirming for the first time the previously published pre-clinical data.^7^ This potential predictive biomarker for gemcitabine efficacy may be of special interest, as additive antibiotic treatment in LPS-positive cases could at least theoretically be used to eliminate bacterial colonisation and thus overcome bacterially mediated chemotherapy resistance as already shown in vivo.^7^ Although gemcitabine monotherapy has been largely replaced by more efficient chemotherapy regimens in the clinically fit patient,^14^ it is still widely used in the clinical practice, especially in patients with significant co-morbidities,^15^ where adjuvant antibiotic treatment in LPS-positive tumours could increase the unfortunately still suboptimal treatment efficacy.

Our study has several limitations that have to be taken into account when interpreting the results: the design was retrospective and a relevant heterogeneity on the applied treatment regimens (specifically in the validation cohort) may lead to a potential bias. In addition, in both cohorts the use of 2nd-line therapy (usually with the alternative cytotoxic backbone, e.g. 5-fluorouracil-based after gemcitabine and vice versa) may have an impact on the results, specifically for OS. However, as the rate of 2nd-line therapy was below 50% in AIO-PK0104, a significant impact of 2nd- and further-line treatments on our results should not be expected. It must also be noted that all patients included in this LPS analysis were treated before the introduction of FOLFIRINOX^16^ or gemcitabine/nab-paclitaxel.^17^ In addition, the LPS immunohistochemistry used only detects a cell wall component of gram-negative bacteria and does not allow assessment of bacterial viability or further characterisation of the bacterial infiltrate. Future studies using more specific methods such as 16s-RNA-FISH or 16s-RNA gene sequencing could complement our rather broad-based approach and specify if it is indeed CDD^L^ expressing bacteria which are associated to worse outcome in gemcitabine- treated patients with LPS-positive tumours. Moreover, although our findings indicate a pivotal role of gram-negative bacteria in mediating gemcitabine resistance, we cannot preclude that we detected a mere surrogate marker, as the impact of the tumour microbiome on chemotherapy resistance may be multifactorial and its effect may not be limited to gemcitabine. For instance, the gram-negative bacteria Fusobacterium nucleatum has been shown to induce resistance to platinum-based chemotherapy in colorectal cancer via autophagy induction.^18^ However, as Fusobacterium nucleatum has a relatively low abundance in PDAC compared to other gram-negative bacteria,^7,13^ a significant impact on the few patients which received platinum compounds in our study is not to be expected.

As both AIO-PK0104 as well as the prospective biomarker study (used for the validation cohort) unfortunately had not collected information about the frequency of endoscopic retrograde cholangiopancreatography (ERCP) including placement of bile duct stents, we are not able to provide data on a potential correlation of the ERCP procedure (and/or the occurrence of cholangitis and subsequent use of antibiotics) in our patient population—aspects that of course would be of additional scientific interest in interpreting the reported data. Future studies on the role of the microbiome in pancreatic cancer should not only focus on potential sources, such as biliary or pancreatic duct instrumentations and the local microbiome in other locations throughout the body, but also on the effect of antibiotic treatments. Thus, examining the microbiome in liquid or solid biopsies may eventually be used to guide informed tumour therapy.

Nevertheless, based on the results from AIO-PK0104 and the validation cohort, intratumoural bacterial LPS detection in PDAC samples may serve as a novel negative predictive biomarker for gemcitabine efficacy, confirming previous pre-clinical observations. These findings must be validated prospectively and should be addressed in the light that this biomarker also may be modifiable by the use of antibiotics.

## Supplementary information

Supplementary files

## Data Availability

All data generated or analysed during this study are included in this published article and its supplementary information file.
