# Correction: Bacterial lipopolysaccharide as negative predictor of gemcitabine efficacy in advanced pancreatic cancer – translational results from the AIO-PK0104 Phase 3 study

**DOI:** 10.1038/s41416-021-01447-1

**Published:** 2021-06-10

**Authors:** Michael Guenther, Michael Haas, Volker Heinemann, Stephan Kruger, Christoph Benedikt Westphalen, Michael von Bergwelt-Baildon, Julia Mayerle, Jens Werner, Thomas Kirchner, Stefan Boeck, Steffen Ormanns

**Affiliations:** 1 Institute of Pathology, Faculty of Medicine, Ludwig-Maximilians-University, Munich, Germany; 2 Department of Internal Medicine III, Grosshadern University Hospital, Ludwig-Maximilians-University, Munich, Germany; 3 German Cancer Consortium (DKTK), partner site Munich, Munich, Germany; 4 Department of Internal Medicine II, Grosshadern University Hospital, Ludwig-Maximilians-University, Munich, Germany; 5 Department of General, Visceral and Transplant Surgery, Ludwig-Maximilians-University, Munich, Germany

**Keywords:** Pancreatic cancer, Predictive markers, Cancer microenvironment, Pancreatic cancer

**Correction to:** *British Journal of Cancer* 10.1038/s41416-020-01029-7, published online 24 August 2020

The article Bacterial lipopolysaccharide as negative predictor of gemcitabine efficacy in advanced pancreatic cancer – translational results from the AIO-PK0104 Phase 3 study, written by Michael Guenther, Michael Haas, Volker Heinemann, Stephan Kruger, Christoph Benedikt Westphalen, Michael von Bergwelt-Baildon, Julia Mayerle, Jens Werner, Thomas Kirchner, Stefan Boeck and Steffen Ormanns, was originally published electronically on the publisher’s internet portal on 24 August 2020 without open access. With the author(s)’ decision to opt for Open Choice the copyright of the article changed on 18 May 2021 to © The Author(s) 2021 and the article is forthwith distributed under a Creative Commons Attribution 4.0 International License, which permits use, sharing, adaptation, distribution and reproduction in any medium or format, as long as you give appropriate credit to the original author(s) and the source, provide a link to the Creative Commons licence, and indicate if changes were made. The images or other third party material in this article are included in the article’s Creative Commons licence, unless indicated otherwise in a credit line to the material. If material is not included in the article’s Creative Commons licence and your intended use is not permitted by statutory regulation or exceeds the permitted use, you will need to obtain permission directly from the copyright holder. To view a copy of this licence, visit http://creativecommons.org/licenses/by/4.0/.

