# Supplementary material for: Bacterial lipopolysaccharide as negative predictor of gemcitabine efficacy in advanced pancreatic cancer – translational results from the AIO-PK0104 Phase 3 study
Source: Br J Cancer. 2020 Aug 24;123(9):1370–6. doi: 10.1038/s41416-020-01029-7 (PMC7591915; doi:10.1038/s41416-020-01029-7)
Supplement: Supplementary file 1 — Supplementary files [file 41416_2020_1029_MOESM1_ESM.pdf]

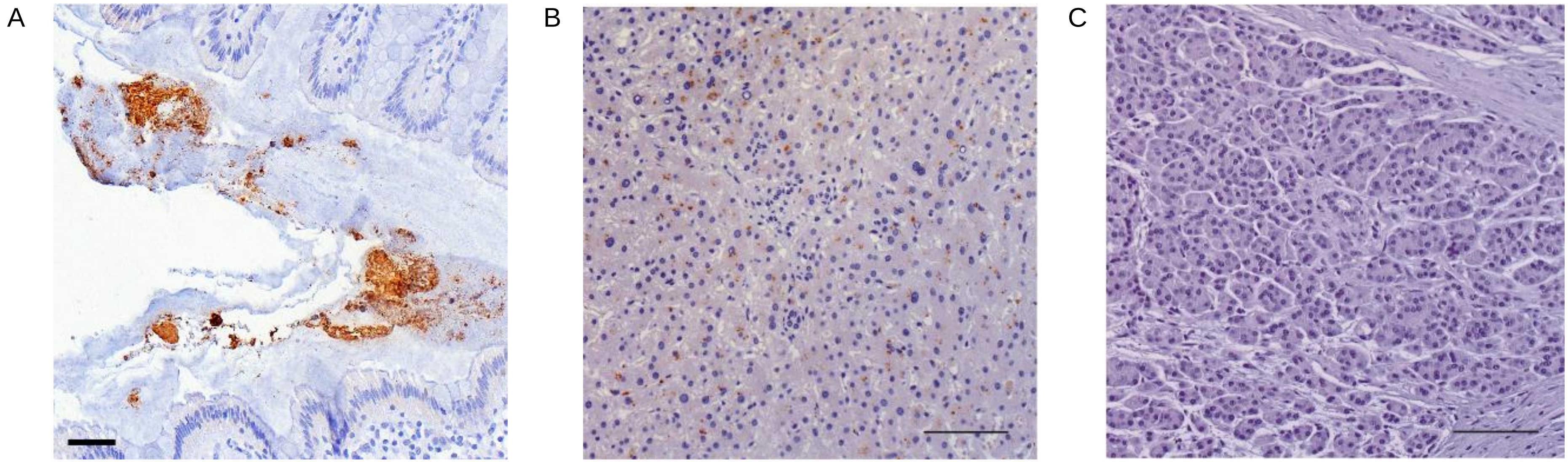

Supplementeray figure S1:

Bacterial lipopolysaccharide (LPS) detection in (A) normal colonic mucosa, (B) normal liver and (C) normal acinar pancreatic tissue

Immunohistochemical detection of bacterial lipopolysaccharide (LPS) in normal colonic mucosa shows distinct and strong a cloud like luminal staining, whereas it appears dot-like and speckled in normal liver. Normal pancreatic tissue shows no staining. 200-fold magnification. Scale bars indicate 50 µm.

		cohort			
		validation cohort	AIO-PK0104	total	p-value (χ2-test)
**disease stage at therapy initiation**	locally advanced	10	23	33	
	metastatic	103	107	210	0.045
total		113	130	243	
**tissue origin**	metastasis	53	73	126	
	primary tumor	60	57	117	0.150
total		113	130	243	
**metastasis at therapy initiation**	lung	6	0	6	
	liver	50	46	96	
	peritonuem	5	7	12	
	other	5	18	23	
	none	10	23	33	
	liver and peritoneum	7	11	18	
	lung and liver	9	2	11	
	liver and bone	0	1	1	0.006
	lung and abdominal lymph nodes	1	2	3	
	liver and lymph nodes	8	14	22	
	liver, lung and lymph nodes	2	2	4	
	lymph nodes	8	3	11	
	liver and adrenal gland	1	1	2	
	lung, liver and bone	1	0	1	
total		113	130	243	
**tissue origin**	hepatic metastasis	46	57	103	
	primary tumor	60	57	117	
	peritoneal metastasis	2	6	8	
	lung metastasis	3	3	6	0.430
	lymph node	1	3	4	
	other / unknown	1	4	5	
total		113	130	243	
**age group**	<=60	46	56	102	
	>60	67	74	141	0.709
total		113	130	243	
**gender**	male	69	76	145	
	female	44	54	98	0.680
total		113	130	243	
**KPS group**	<= 80	51	52	103	
	> 80	62	76	138	0.480
total		113	128	241	
**CTX type**	gemcitabine 1st line subgroup	19	59	78	
	non-gemcitabine 1st line subgroup	94	71	165	0.000
total		113	130	243	
**grade group**	G1-G2	51	38	89	
	G3-G4	62	92	154	0.010
total		113	130	243	

Supplementary table S1:
Comparison of the clinicopathologic parameters in the AIO-PK0104 and the validation cohort

			n	OS (months)	p (log-rank)	HR	95% CI	PFS (months)	p (log-rank)	HR	95% CI
AIO-PK0104	sex	male	76	5.3	0.614	0.910	0.631 - 1.313	2.9	0.861	0.969	0.676 - 1.387
		female	54	7.3				2.7			
	age group	< 60 years	56	5.8	0.565	0.899	0.624 - 1.294	2.5	0.449	0.872	0.610 - 1.246
		≥ 60 years	74	6.7				2.9			
	KPS	≤ 80	52	3.4	0.000	0.412	0.282 - 0.601	2.1	0.009	0.616	0.425 - 0.892
		> 80	76	8.2				3.7			
	1st line CTX type	capecitabine-based	59	6.7	0.323	1.201	0.834 - 1.731	2.2	0.039	0.686	0.479 - 0.984
		gemcitabine-based	71	5.8				3.6			
	tumor grade	G1 - G2	38	9.6	0.015	1.644	1.096 - 2.465	3.7	0.012	1.651	1.112 - 2.452
		G3 - G4	92	5.7				2.5			
	tumor stage	locally advanced	23	10.6	0.007	1.932	1.187 - 3.145	4.0	0.109	1.449	0.916 - 2.291
		metastatic	107	5.7				2.7			
validation cohort	sex	male	69	9.1	0.672	0.916	0.610 - 1.376	7.0	0.628	1.124	0.701 - 1.801
		female	44	8.5				4.7			
	age group	< 60 years	46	8.3	0.385	1.197	0.797 - 1.800	6.6	0.776	1.070	0.670 - 1.708
		≥ 60 years	67	8.6				6.3			
	KPS	≤ 80	51	6.7	0.022	0.625	0.415 - 0.939	4.6	0.066	0.644	0.402 - 1.033
		> 80	62	11.8				7.8			
	1st line CTX type	non gemcitabine-based	19	9.1	0.014	0.523	0.310 - 0.885	2.7	0.000	0.358	0.199 - 0.645
		gemcitabine-based	94	8.5				7.6			
	tumor grade	G1 - G2	51	12.4	0.017	1.622	1.084 - 2.427	9.2	0.060	1.565	0.978 - 2.504
		G3 - G4	62	7.0				5.8			
	tumor stage	locally advanced	10	18.8	0.059	2.076	0.957 - 4.503	-	0.047	2.686	0.974 - 7.412
		metastatic	103	8.4				6.3			
all cases	sex	male	145	6.8	0.525	0.916	0.699 - 1.201	4.0	0.773	1.043	0.785 - 1.385
		female	98	8.3				3.6			
	age group	< 60 years	102	7.3	0.710	1.053	0.803 - 1.380	3.7	0.969	0.994	0.750 - 1.318
		≥ 60 years	141	7.9				3.8			
	KPS	≤ 80	103	4.4	0.000	0.556	0.422 - 0.731	2.8	0.003	0.646	0.485 - 0.862
		> 80	138	9.2				4.7			
	1st line CTX type	non gemcitabine-based	78	6.9	0.041	0.743	0.558 - 0.989	2.2	0.000	0.448	0.332 - 0.606
		gemcitabine-based	165	8.0				5.3			
	tumor grade	G1 - G2	89	10.5	0.000	1.734	1.309 - 2.297	6.2	0.000	1.801	1.337 - 2.426
		G3 - G4	154	6.2				3.2			
	tumor stage	locally advanced	33	13.2	0.014	1.644	1.103 - 2.450	5.3	0.136	1.361	0.905 - 2.046
		metastatic	210	7.0				3.7			

Supplementary table S2: *Patient prognosis according to clinicopathologic variables in each patient subgroup*

Median times of overall survival (OS) and progression free survival (PFS) according to clinicopathologic variables in each study subgroup as well as corresponding hazard ratios (HR) and 95% confidential intervals (95% CI).

	case #	LPS	
		TMA	whole mount
**AIO-PK0104**	18	+	+
	21	+	+
	34	+	+
	78	+	+
	89	+	+
**validation cohort**	16	+	+
	17	+	+
	32	-	-
	36	-	-
	48	-	-
	51	-	-
	53	-	-
	60	-	-
	62	-	-
	67	-	-
	68	-	-
	70	-	-
	71	-	-
	118	+	+
	144	+	+
	165	+	+
	174	-	-
	183	-	-
	185	-	-
	186	-	-

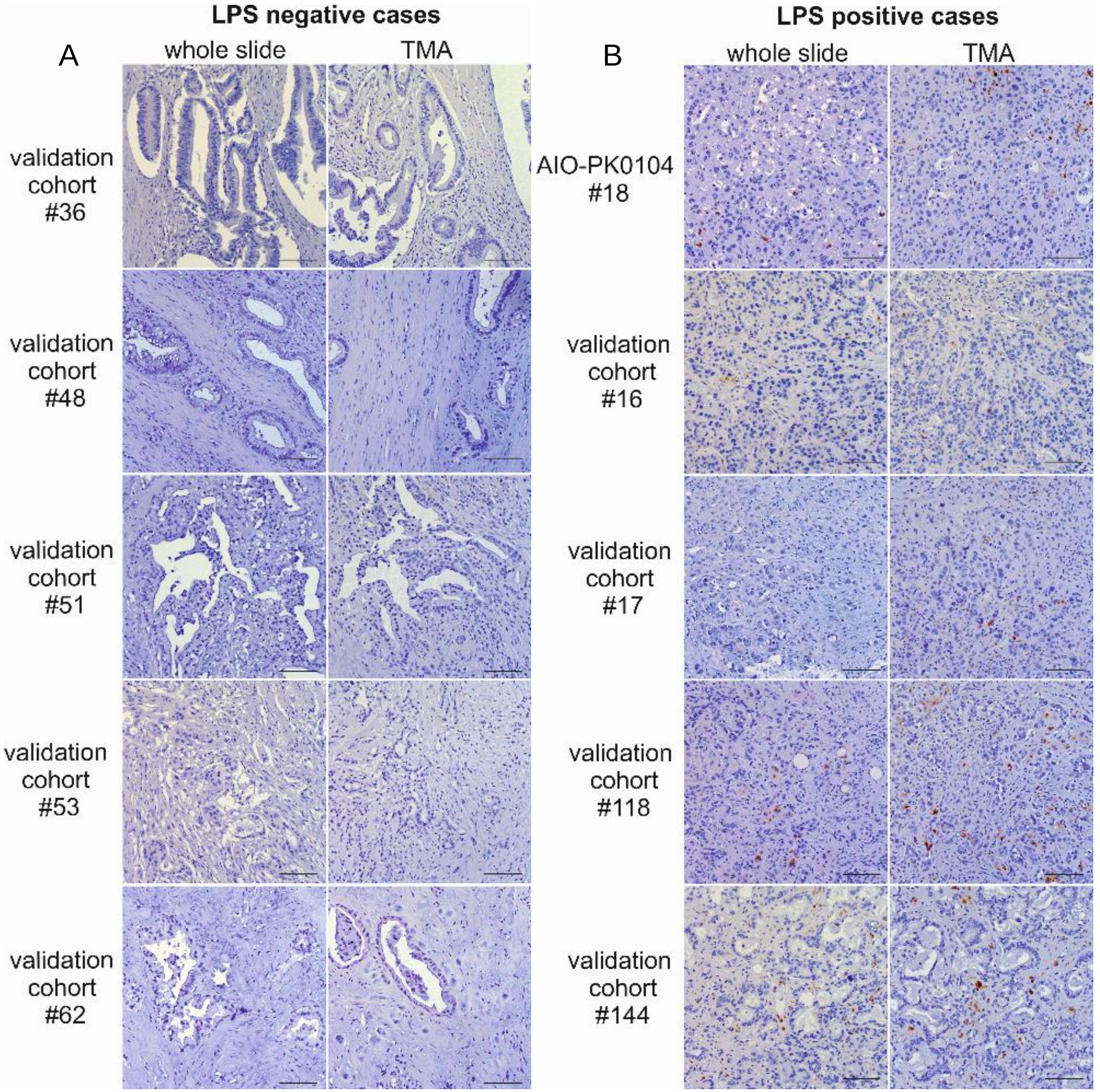

Supplementary table S3 and supplementary figure S2:

Complete concordance of LPS detection in TMA cores and corresponding whole mount tissue sections

Comparison of the staining results in TMA tissue and whole mount tissue sections of ten exemplary positive and 15 exemplary negative PDAC cases, of which five negative cases are depicted in figure S2 A and five positive cases in figure S2 B.

		LPS -	LPS +	total	p-value (χ^2-test)
primary tumor location	unknown	10	2	12	0.484
	pancreatic head	59	5	64	
	pancreatic body	21	1	22	
	pancreatic tail	16	3	19	
total		106	11	117	

Supplementary table S4: *LPS detection is not associated to primary tumor location*

Cross tabulation of LPS positivity in PDAC primary tumor samples according to the primary tumor location.

		**LPS**	**n**	**%**	**OS** (months)	**p** (log-rank)	**HR**	**95% CI**	**PFS** (months)	**p** (log-rank)	**HR**	**95% CI**
primary tumor	total	+	11	9.4	2.6	0.035	1.619	0.806 - 3.253	1.6	0.116	1.661	0.887 - 3.149
		-	106	90.6	11.5				4.7			
	gemcitabine 1st line subgroup	+	10	12.8	1.6	0.016	1.748	0.821 - 3.720	1.6	0.030	2.123	1.058 - 4.262
		-	68	87.2	15.4				7.8			
	non-gemcitabine 1st line subgroup	+	1	2.6	5.2	0.349	2.555	0.332 - 19.668	0.9	0.001	17.994	1.631 - 198.467
		-	38	97.4	9.4				2.5			
metastatic tissue	total	+	46	38.1	6.9	0.096	1.471	1.000 - 2.164	3.6	0.526	1.138	0.762 - 1.699
		-	80	61.9	7.3				3.2			
	gemcitabine 1st line subgroup	+	33	37.9	6.4	0.042	1.873	1.170 - 2.999	3.7	0.407	1.225	0.757 - 1.984
		-	54	62.1	8.3				3.8			
	non-gemcitabine 1st line subgroup	+	13	33.3	7.2	0.781	0.945	0.466 - 1.916	3.3	0.899	0.954	0.460 - 1.980
		-	26	66.7	4.9				2.2			

Supplementary table S5: *Similar effect of LPS detection on PFS and OS in metastatic and primary tumor tissue origin subgroups*

Median times of overall survival (OS) and progression free survival (PFS) according to intratumoral LPS detection in each subgroup according to tumor tissue origin (primary tumor vs metastatic tissue) as well as corresponding hazard ratios (HR) and 95% confidential intervals (95% CI).

		LPS	n	%	OS (months)	p (log-rank)	HR	95% CI	PFS (months)	p (log-rank)	HR	95% CI
TMA	total	+	16	12.0	6.4	0.017	1.896	1.089 - 3.302	3.2	0.085	1.638	0.928 - 2.890
		-	117	88.0	9.6				4.6			
	gemcitabine 1st line subgroup	+	12	12.9	6.4	0.012	2.266	1.177 - 4.364	3.7	0.143	1.651	0.837 - 3.257
		-	81	87.1	11.7				6.6			
	non-gemcitabine 1st line subgroup	+	4	10.0	5.7	0.695	1.169	0.408 - 3.352	1.5	0.042	2.899	0.989 - 8.496
		-	36	90.0	7.3				2.7			
whole mount tissue	total	+	41	37.3	5.6	0.027	1.586	1.044 - 2.410	3.5	0.864	1.038	0.679 - 1.587
		-	69	32.7	7.9				3.2			
	gemcitabine 1st line subgroup	+	31	43.1	5.6	0.022	1.733	1.044 - 2.879	3.7	0.216	1.389	0.823 - 2.344
		-	41	56.9	7.9				4.6			
	non-gemcitabine 1st line subgroup	+	10	26.3	6.9	0.496	1.463	0.668 - 3.206	3.3	0.247	0.618	0.270 - 1.411
		-	28	73.7	6.7				1.9			

Supplementary table S6: *Similar effect of LPS detection on PFS and OS in whole mount tissue sections and TMA based tissue*

Median times of overall survival (OS) and progression free survival (PFS) according to intratumoral LPS detection in each subgroup according to tissue modality (TMA vs whole mount tissue) as well as corresponding hazard ratios (HR) and 95% confidential intervals (95% CI).

randomization arm	LPS	PD	SD/PR/CR	DCR (%)	p-value (χ^2)
capecitabine 1st line	+	6	4	40.0	0.589
	-	23	17	42.5	
gemcitabine 1st line	+	4	8	66.7	0.534
	-	18	30	62.5	

Supplementary table S7: *LPS detection is not associated with radiological tumor response in the AIO-PK0104 cohort according to RECIST criteria*

Disease control rates (DCR) according to LPS positivity in each randomization arm of 110 patients from the AIO-PK0104 trial. PD = progressive disease, SD = stable disease, PR = partial response, CR = complete remission.
